# Combined Preimplantation Genetic Testing for Genetic Kidney Disease: Genetic Risk Identification, Assisted Reproductive Cycle, and Pregnancy Outcome Analysis

**DOI:** 10.3389/fmed.2022.936578

**Published:** 2022-06-17

**Authors:** Min Xiao, Hua Shi, Jia Rao, Yanping Xi, Shuo Zhang, Junping Wu, Saijuan Zhu, Jing Zhou, Hong Xu, Caixia Lei, Xiaoxi Sun

**Affiliations:** ^1^Shanghai Ji Ai Genetics and IVF Institute, The Obstetrics and Gynecology Hospital of Fudan University, Shanghai, China; ^2^Department of Nephrology, Children’s Hospital of Fudan University, National Pediatric Medical Center of China, Shanghai, China; ^3^Shanghai Key Laboratory of Female Reproductive Endocrine-Related Diseases, The Obstetrics and Gynecology Hospital of Fudan University, Shanghai, China

**Keywords:** preimplantation genetic testing, monogenic kidney disease, haplotype analysis, pregnancy outcomes, expanded carrier screening

## Abstract

**Background:**

Genetic kidney disease is a major cause of morbidity and mortality in neonates and end-stage renal disease (ESRD) in children and adolescents. Genetic diagnosis provides key information for early identification of congenital kidney disease and reproductive risk counseling. Preimplantation genetic testing for monogenic disease (PGT-M) as a reproductive technology helps prospective parents to prevent passing on disease-causing mutations to their offspring.

**Materials and Methods:**

A retrospective cohort of couples counseled on PGT who had a risk to given birth to a child with genetic kidney disease or had a history of prenatal fetal kidney and urinary system development abnormalities from 2011 to 2021. Through a combination of simultaneously screening for aneuploidy and monogenic kidney disease, we achieved reproductive genetic intervention.

**Results:**

A total of 64 couples counseled on PGT for monogenic kidney disease in a single reproductive center during the past 10 years, of whom 38 different genetic kidney diseases were identified. The most frequent indications for referral were autosomal recessive disease (54.7%), then autosomal dominant disease (29.7%), and X-linked disease (15.6%). Polycystic kidney disease was the most common diseases counted for 34.4%. After oocyte-retrieval in all of 64 females, a total of 339 embryos were diagnosed and 63 embryos were transferred in succession. Among 61 cycles of frozen-embryo transfer (FET), ongoing pregnancy/live birth rate (OP/LBR) reached 57.38%. The cumulative OP/LBR in our cohort for the 64 couples was 54.69%. In addition, we have carried out expanded carrier screening (ECS) in all the *in vitro* fertilization (IVF) couples performed PGT covering 7,311 individuals. The carrier frequency of the candidate genes for monogenic kidney diseases accounted for 12.19%.

**Conclusion:**

Overall, the customization PGT-M plan in our IVF center is pivotal to decreasing the morbidity and implementing reproductive genetic intervention of genetic kidney disease.

## Introduction

Genetic kidney disease is a major cause of chronic kidney disease (CKD) in children, resulting in substantial morbidity and mortality as well as the high healthcare costs (1). Genetic kidney disease includes congenital anomalies of the kidney and urinary tract (CAKUT), congenital nephrotic syndrome, renal tubular diseases and cystic kidney disease. Renal phenotypes are also associated with many syndromic disorders and rare diseases. A number of individuals with genetic kidney disease failed to get a precise diagnosis until developing into renal failure. Genetic diagnosis for kidney disease enables counseling for all the affected pedigree members on prognosis and therapeutic options, as well as family risk counseling and planning (2, 3).

Preimplantation genetic testing (PGT) is one assisted reproductive technology (ART) available to individuals who carry a disease-causing genetic variant. PGT collects the embryo’s genetic material for genetic analysis to identify healthy embryo prior to frozen- embryo transfer (FET). PGT is conducted as part of an *in vitro* fertilization (IVF) cycle and requires embryo biopsy, which may undertake at the cleavage stage (day 3 of development) with the removal of 1–2 cells or now more commonly carried out at the blastocyst stage (day 5–7 of development) with the removal of up to 10 cells (4). This approach thereby greatly reduces the chance of having a pregnancy affected with the genetic disease. Since the initial practice of PGT in the monogenetic disorders in 1990s (5), it has been extensively employed in the diagnosis of monogenic disease, X-linked disorders, aneuploidy, and chromosomal rearrangements (3, 6–10). Carrier screening is becoming standard practice for egg and sperm donors and couples seeking assisted reproduction, due to the introduction of target panels that screen for multiple variants in low risk populations to detect carriers of single-gene disorders (11). There have been over 500 disorders in which PGT-M has been successfully applied across the world (12). Several studies have reported the patient series with PGT for genetic kidney disease (4, 5, 11). Advances in molecular genetics have made a great impact on PGT application in China, whereas few studies reported the clinical outcome of PGT and the gene frequency of genetic kidney disease through carrier screening.

This study focused on combined PGT (PGT-M/A/SR) for monogenetic kidney disease at a single medical center from 2011 to 2021. The retrospect analysis on the clinical features, genotypes of PGT, pregnancy rate and outcomes were performed to provide clinical suggestions and decision making for PGT with monogenetic kidney disease.

## Materials and Methods

### Study Design and Participants

The local ethics committee of Shanghai JIAI Genetics and IVF Institute approved and supervised this retrospective cohort study, adhering to the Declaration of Helsinki. Prior to enrolling individuals in the PGT cycles, written informed permissions from all the participants were obtained. All the participants were retrospectively recruited between January 2011 and December 2021 at Shanghai JIAI Genetics and IVF Institute. The eligibility enrollment included couples at risk for reproductive genetic kidney disease with comprehensive phenotype, genotype, karyotype, gestation history, family history, pedigree information, and/or the outcome of pregnancy. ECS genotype information including variants of known monogenetic disease causative genes (Supplementary Table 1) were collected to evaluated of risk for monogenic conditions. And we also collected all of records of the pregnancies that the couples who had counseled on PGT. Exclusion criteria were oocytes or sperm from a donor source which led to changes in the genetic probability of the genetic kidney disease and incomplete records of the above information or the couples who transmitted to other medical centers after consulting.

### Clinical Process of Preimplantation Genetic Testing and *in vitro* Fertilization Cycle

**FIGURE 1:**
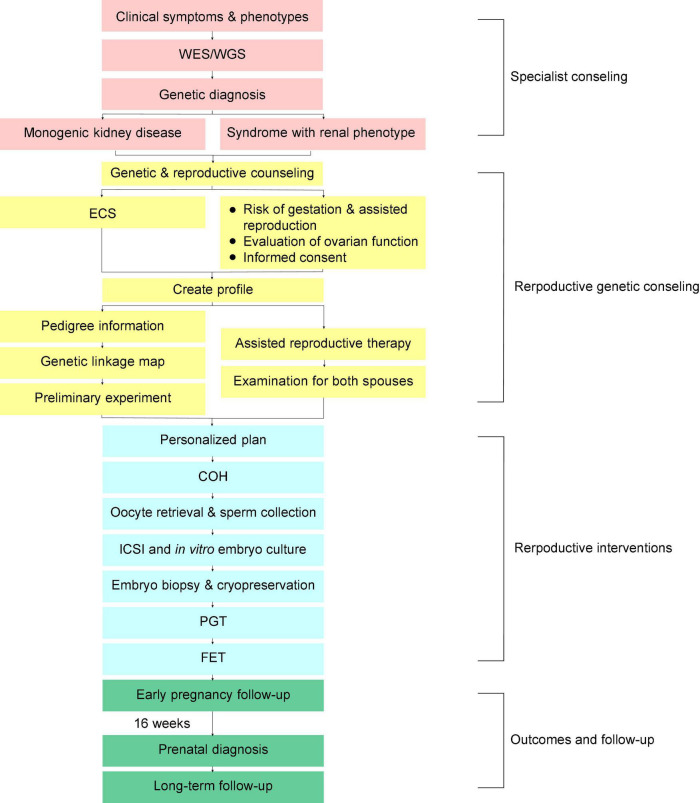
Flowchart showing the preimplantation genetic testing for monogenic disease (PGT-M) procedures and follow-up of couples with genetic kidney disease. WES/WGS, whole exome sequencing/whole genome sequencing; ECS, expanded carrier screening; ICSI, intracytoplasmic sperm injection; COH, controlled ovarian hyperstimulation; PGT, preimplantation genetic testing; FET, frozen-embryo transfer.

The PGT process at our center is shown in the flowchart (Figure 1). Consultants transferred from the specialties should fetch their genetic test results or go through the WES/WGS depending on the clinical assessments of the clinical geneticists and the nephrologists. Variant interpretation was performed manually by a panel of nephrologists and clinical molecular geneticists. The final diagnosis was confirmed according to the combination of clinical manifestations, pedigree verification and the genetic test results. The variants were classified according to the American College of Medical Genetics and Genomics (ACMG) guidelines (13). Besides the causative genes, other locus with possibility of pathogenesis was checked in expanded carrier screening (ECS) (11). After final diagnosis and ECS, a PGT-M scheme was designed by the doctors with consent of consultants, and the genetic linkage map was built.

Examination was needed for both males and females before making a personalized plan of oocyte retrieval. The ovarian stimulation for IVF included long gonadotrophic releasing hormone (GnRH) agonist, short GnRH agonist, antagonist, and mild stimulation protocols. About one and a half days after triggering with human chorionic gonadotropin (hCG) or an agonist, the oocyte was retrieved under transvaginal ultrasound guidance. Intracytoplasmic sperm injection (ICSI) was applied to all PGT-IVF schemes along with the FET. The embryos were washed and cultured to develop to the blastocyst stage interspersed with making an 18-μm hole in the zona pellucida on day 4 (D4) after fertilization. Blastocysts with trophectoderm (TE) cells were chosen for biopsy on D5 or D6. In general, 3–8 biopsied TE cells were used directly for whole genome amplification (WGA) followed by karyomapping. All experiments and data analysis were performed in the JIAI local laboratory. The prenatal diagnosis was performed at about the 16th week after FET. All the above procedures adopt the standard IVF techniques of Shanghai JIAI Genetics and IVF Institute (14).

### Expanded Carrier Screening

Expanded carrier screening was routinely offered as an option to all patients seeking PGT in JIAI center between 1 May 2017 and 31 December 2021. The panel covers 213 genes implicated in 147 recessive (autosomal or X-linked) diseases in order to help couples to further reduce the risk of childbearing children with other recessive genetic diseases. The ECS assay used Agilent custom capture probe kit, Illumina Cluster and SBS kit. Based on Illumina platform (Illumina, San Diego, CA, United States^1^), high-throughput sequencing was performed on exons and introns within ± 10 bp of 213 genes in the genomic DNA of the subjects. The sequencing results were compared with human reference gene sequences, and all sequenced fragments were identified by bases. In this test, the target region capture high-throughput sequencing technology was adopted, and only the target gene coding region was sequenced, with an average coverage of 110–160×.

Sentieon software suite was used to analyze sequencing data. The sequencing fragments were compared with the Sentieon BWA and UCSC Hg19 reference genome. Variations are annotated by VEP software (Variant Effect Predictor) (15). Three major databases of known or suspected pathogenic variants, including ClinVar, OMIM, and HGMD, will be used to screen known pathogenic variants, as well as multiple tools for predicting missense variants and annotation of non-coding regulatory sequences. Population-based large-scale sequencing databases (gnomAD V2.1.1) were used to exclude mutations with a high frequency in the normal population (16).

Each variation in this assay will be evaluated using Clinical Sequence Analyzer (CSA) software (Mingma Technologies, Shanghai, China). After optimization according to the Sequence Variation Interpretation Standards and Guidelines published by ACMG and the latest guidelines developed by ClinGen, each variation was curated (17–19). This test reports only those mutations curated as pathogenic or likely pathogenic (P/LP) in the exon and intron segments within ±10 bp of the genes contained in the schedule. Standard Human Genome Variation Society recommendations (HGVS Nomenclature v15.11) nomenclature was used for sequence variation (20).

The copy number variation (CNV) analysis tool based on high-throughput sequencing was used to detect CNV, including specific CNV in *DMD*, *SMN1*, *HBA1*, and *HBA2* genes of the subjects, and the suspected positive samples were verified by multi-junction probe amplification technology and capillary electrophoresis technology. Coffalyser.Net software (MRC-Holland, Amsterdam, Netherlands^2^) was used for quality control and data analysis.

### Genetic Testing of Blastocyst

Whole genome amplification was performed using a REPLI-g Single Cell Kit (QIAGEN, Hilden, Germany) according to the manufacturer’s protocol as described earlier (14). For tissue and peripheral blood samples from the family, the DNA was isolated using DNeasy Blood & Tissue Kit as described in the manufacturers’ protocol (QIAGEN, Germany). The single nucleotide polymorphism (SNP) genotype and intensity of the WGA products and DNA samples from peripheral blood or tissue were determined with an Illumina HumanKaryomap-12 v1.0 microarray for PGT-M (Illumina, Inc., San Diego, CA, United States). Each bead chip can simultaneously analyze approximately 300,000 SNP loci (Illumina, Inc. San Diego, CA, United States). The SNP array experiments followed the reported standardized procedures in accordance with the infinium chip protocol. Then the BeadChips were imaged on an iScan System (Illumina, Inc., San Diego, CA, United States). The microarray scanning results were processed using the B allele frequency and Log R Ratio. The software used for subsequent analysis and the analysis process are as previously reported (14). The Karyomapping data for each sample was imported into BlueFuse-Multi V4.0 software (Illumina, San Diego, CA, United States) according to the analysis guidelines (21) recommended by the manufacturer for genome wide analysis of genetic disease based on SNP haplotyping mapping crossovers between parental haplotypes to distinguish the chromosomes that carried the mutation.

As in Supplementary Table 2, embryos can be classified as euploid, aneuploid and low-level mosaicism according to chromosomal abnormalities. Mosaicism referred to the occurrence of two or more genomes in an individual/embryo derived from a single zygote, including whole-chromosome and CNV-level mosaicism (22, 23). The low-level mosaicism (≤50%) referred to the simple mosaicism of a single chromosome or a segmental chromosome. The proportion of abnormal mosaicism was less than 50% and the simple low-level mosaicism could be the secondary candidates of FET (14). Euploid embryos were sub-classified into three categories: the unaffected embryos refer to the embryos without paternal or maternal variants; the affected embryos refer to those with variants from both paternal and maternal in AR diseases, at least one variants of dominant diseases associated gene, or the hemizygous variants of X-linked recessive diseases associated gene; the embryos of carrier embryos refer to the one which carried only one paternal/maternal variants in AR diseases or the heterozygote variants of X-linked recessive diseases.

### Primary Outcomes, Embryo Transfer, Antenatal Examination, and Antenatal Genetic Diagnosis

Pregnancy outcomes were primarily observed as positive-human chorionic gonadotropin (PHCG), implantation rate (IR), fetal heartbeat (FHB), ongoing pregnancy/live birth rate (OP/LBR), biochemical pregnancy rate (BPR), and spontaneous abortion (SAB) rate (14). Cumulative OP/LBR were also calculated, which were the percentage of all couples who underwent IVF cycles that ultimately had at least one OP/LBR.

Under ultrasound guidance, embryos free of disease-causing genes and chromosome abnormalities were transferred. After that, 5 early pregnancy follow-up visits will be conducted in our IVF center, and the birth follow-up will be conducted by telephone and relevant indicators will be registered as previously published (14). The prenatal examination was performed with ultrasonography throughout the gestations of our consultants. In the second trimester pregnancy, the invasive prenatal genetic diagnosis was performed using chorionic villus sampling (starting at about the 12th week of pregnancy) or amniocentesis (starting at about the 16th week of pregnancy).

### Statistical Analysis

All the statistical analysis was performed with MS Excel and SPSS (version 25.0, IBM, Armonk, NY, United States). Graph was created using GraphPad prism 8.3.0. Statistical significance was defined as a *P*-value of less than 0.05.

## Results

### Clinical Characteristics

Between 2011 and 2021, we analyzed a total of 463 cases (8.0%) for PGT-M from the 5,770 couples who received fertility and reproductive counseling at a signal medical center in Shanghai, China. There were 64 couples who were diagnosed with pathogenic/likely pathogenic (P/LP) variants of known disease causative genes for kidney disease or the syndromes with renal phenotypes, which accounted for 13.8% (64/463) of all PGT-M (Table 1).

**TABLE 1:** Baseline characteristics of preimplantation genetic testing for monogenic disease (PGT-M) couples in the genetic kidney diseases/syndromes with renal phenotypes cohort.

Characteristic	Total couples, *n* = 64
**Clinical information**	**Year, mean ± SD (range)**
Maternal age at first counseling	30.3 ± 4.1 (23–42)
Paternal age at first counseling	33.5 ± 4.8 (26–44)
**Diseases of the proband**	***n* (%)**
Renal disease	44 (68.8)
Syndrome with renal phenotypes	20 (31.3)
**Complains**	***n* (%)**
Abnormal gestation	29 (45.3)
Birth defect	27 (42.2)
Family history of genetic kidney disease	17 (26.6)
Infertility	10 (15.6)
**PGT-M**	
Number of females undergo oocyte-retrieval (*n*, %)	64 (100)
Number of females undergo IVF-FET (*n*, %)	43 (67.2)
Age at oocyte-retrieval (years ± SD, range)	31.3 ± 4.1 (24–42)
Maternal age at oocyte-retrieval ≥ 35 years	15.6% (10/64)
Maternal age at IVF-FET (years ± SD, range)	30.6 ± 3.0 (25–37)
Paternal age at IVF-FET (years ± SD, range)	34.4 ± 5.1 (26–47)
Maternal age at IVF-FET ≥ 35 years	9.3% (4/43)

*FET, frozen-embryo transfer; IVF, in vitro fertilization; PGT-M, preimplantation genetic testing for monogenic disease.*

**FIGURE 2:**
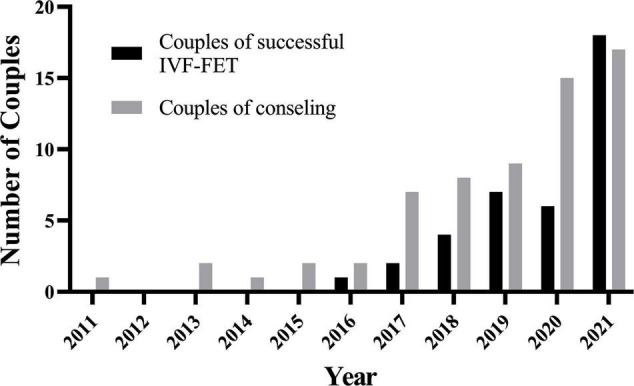
The number of couples of consulting/successful IVF-FET has grown since 2018 in one medical center. IVF-FET, *in vitro* fertilization and frozen-embryo transfer.

Between 2011 and 2016, our center counseled less than five couples with kidney disease every year. It has grown since 2018, reaching 17 occurrences in 2021 (Figure 2). Cumulatively, 35 couples have been conceived through IVF-FET from 2016 to end 2021. Maternal age was 2 years younger than paternal age at the time of first counseling, with a mean age of 30.3 years.

Among the 64 couples who had identified the P/LP variants of known kidney disease, the main reasons for their ART requests included abnormal pregnancy (29, 45.3%), birth defect (27, 42.2%) with some overlaps of abnormal pregnancy (8). Only 17 (26.6%) couples had a positive family history of kidney disease (Table 1 and Supplementary Figure 1).

### Genetic Diagnosis for Monogenetic Kidney Disease

**FIGURE 3:**
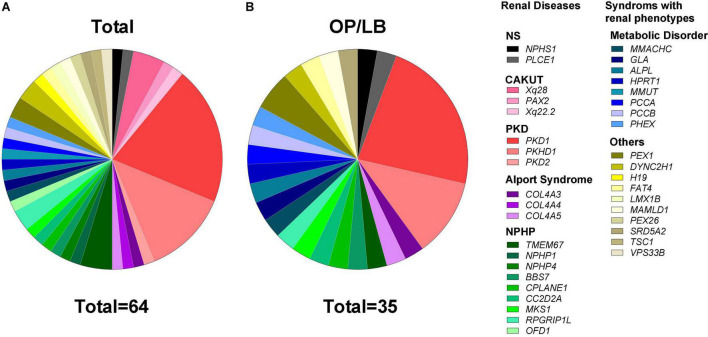
Proportion of cases with diagnosis of pathogenic/likely pathogenic variants in genetic kidney disease. **(A)** Genetic spectrum of kidney disease in 64 couples who were diagnosed with pathogenic/likely pathogenic variants of known disease causative genes for kidney disease or the syndromes with distinct renal phenotypes. **(B)** The gene distribution of 35 couples with ongoing pregnancy/live birth (OP/LB) by the end of 2021. CAKUT, congenital anomalies of the kidney and the urinary tract; NPHP, nephronophthisis; NS, nephrotic syndrome; PKD, polycystic kidney disease.

Genetic findings for the 64 couples identified the P/LP variants in 38 known disease-causing genes for kidney disease and clinical outcomes were also listed (Figure 3A, Table 2, and Supplementary Table 3). The P/LP variations founded in 20 monogenetic diseases were carried by 44 couples (68.8%), whereas P/LP variations in 18 syndromic diseases with both renal and extrarenal phenotypes were carried by 20 couples (31.3%). There were 35 couples (54.7%) with diagnosis of autosomal recessive (AR) disease (Supplementary Table 4), 19 couples (29.7%) with a high risk for autosomal dominant (AD) disease and 10 (15.6%) with diagnosis of X-linked disease. Polycystic kidney disease (PKD) was the most common disease in our cohort accounted for 34.4%, followed by nephronophthisis (NPHP, 18.8%), metabolic disease (12.5%) and congenital anomalies of the kidney and urinary tract (CAKUT, 7.8%).

**TABLE 2:** Genes and variants list in the preimplantation genetic testing for monogenic disease (PGT-M) cohort of genetic kidney diseases/syndromes with renal phenotypes cohort.

ID	Affected gene	Mutation type	Transcript	Map location	DNA change	Amino acid change	Paternal (P)/ Maternal (M)	Clinical outcomes
1	*ALPL*	Missense	NM_000478.6	*1p36.12*	c.212G>A	p.Arg71His	M	LB
2	*BBS7*	Missense	NM_176824.3	*4q27*	c.634T>C	p.Ser212Pro	P	OP
	*BBS7*	Missense	NM_176824.3	*4q27*	c.849 + 1G>C	−	M	
3	*CC2D2A*	Missense	NM_001080522.2	*4p15.32*	c.2728C>T	p.Arg910Ter	M	LB
	*CC2D2A*	Missense	NM_001080522.2	*4p15.32*	c.4598T>C	p.Leu1533Pro	P	
4	*COL4A3*	Missense	NM_000091.5	*2q36.3*	c.1918G>A	p.Gly640Arg	M	LB
5	*COL4A5*	Missense	NM_000495.5	*Xq22.3*	c.619G>C	p.Gly207Arg	P & M	OP
6	*CPLANE1*	Missense	NM_023073.3	*5p13.2*	c.7978C>T	p.Arg2660Ter	P	LB
	*CPLANE1*	Missense	NM_023073.3	*5p13.2*	c.3830G>A	p.Cys1277Tyr	M	
7	*DYNC2H1*	Missense	NM_001080463.2	*11q22.3*	c.7594C>T	p.Arg2532Trp	P	Implantation failure
	*DYNC2H1*	Missense	NM_001080463.2	*11q22.3*	c.5176C>T	p.Arg1726Ter	M	
8	*DYNC2H1*	Missense	NM_001080463.2	*11q22.3*	c.10163C>T	p.Pro3388Leu	P	Frozen
	*DYNC2H1*	Missense	NM_001080463.2	*11q22.3*	c.7574A>C	p.Glu2525Ala	P	
9	*DYNC2H1*	Missense	NM_001080463.2	*11q22.3*	c.10163C>T	p.Pro3388Leu	P	OP
	*DYNC2H1*	Missense	NM_001080463.2	*11q22.4*	c.703T>C	p.Trp235Arg	M	
10	*FAT4*	Missense	NM_024582.5	*4q28.1*	c.10261A>C	p.Thr3421Pro	P	OP
	*FAT4*	Missense	NM_024582.5	*4q28.1*	c.5191C>T	p.Gln1731*	M	
11	*GLA*	Missense	NM_000169.2	*Xq22.1*	c.888G>A	p.Met296Ile	M	LB
12	*H19*	Gross deletion	NR_002196.2	*11p15.4*	H19-up-214nt, H19-up-454nt	−	M	Frozen
13	*HPRT1*	Missense	NM_000194.3	*Xq26.2-q26.3*	c.533-9T>G	−	M	OP
14	*LMX1B*	Small deletion	NM_001174146.2	*9q33.3*	c.712_714del	p.Phe238del	M	Implantation failure
15	*MAMLD1*	Missense	NM_001177465.3	*Xq28*	c.2398C>T	p.Gln800Ter	M	LB
16	*MKS1*	Repeat variants	NM_017777.4	*17q22*	c.1411dup	p.Glu471GlyfsTer120	P & M	LB
17	*MMACHC*	Missense	NM_015506.3	*1p34.1*	c.609G>A	p.Trp203Ter	M	LB
	*MMACHC*	Small deletion	NM_015506.3	*1p34.1*	c.656_658del	p.Lys220del	P	
18	*MMUT*	Missense	NM_000255.4	*6p12.3*	c.1106G>A	p.Arg369His	P	Frozen
	*MMUT*	Missense	NM_000255.4	*6p12.3*	c.914T>C	p.Leu305Ser	M	
19	*NPHP1*	Gross deletion	NM_000272.5	*2q13*	exon1-20del	−	P & M	Frozen
20	*NPHP4*	Small deletion	NM_015102.5	*1p36.31*	c.3122del	p.Phe1041SerfsTer42	P	Frozen
	*NPHP4*	Small deletion	NM_015102.5	*1p36.31*	c.169_176del	p.His57SerfsTer5	M	
21	*NPHS1*	Missense	NM_004646.4	*19q13.12*	c.3478C>T	p.Arg1160Ter	M	LB
	*NPHS1*	Missense	NM_004646.4	*19q13.12*	c.2663G>A	p.Arg888Lys	P	
22	*OFD1*	Small deletion	NM_003611.3	*Xp22.2*	c.2del	p.Met1ArgfsTer14	M	Frozen
23	*PAX2*	Missense	NM_003987.5	*10q24.31*	c.254G>A	p.Gly85Asp	M	Frozen
24	*PCCA*	Small deletion	NM_000282.4	*13q32.3*	c.376del	p.Ala126Profs*58	M	LB
	*PCCA*	Missense	NM_000282.4	*13q32.3*	c.809T>C	p.Ile270Thr	P	
25	*PCCB*	Non-sense	NM_001178014.1	*3q22.3*	c.184-2A>G	−	P	LB
	*PCCB*	Missense	NM_001178014.1	*3q22.4*	c.331C>T	p.Arg111Ter	M	
26	*PEX1*	Missense	NM_000466.3	*7q21.2*	c.1483 + 1G>A	−	P	LB
	*PEX1*	Repeat variants	NM_000466.3	*7q21.2*	c.1725dup	p.Arg577ThrfsTer15	M	
27	*PEX1*	Repeat variants	NM_000466.3	*7q21.2*	c.892_895dup	p.Asn299IlefsTer2	M	LB
	*PEX1*	Small deletion	NM_000466.3	*7q21.2*	c.2927-2del	−	P	
28	*PEX26*	Small deletion	NM_017929.6	*22q11.21*	c.34del	p.Leu12SerfsTer70	P	Frozen
	*PEX26*	Small deletion	NM_017929.6	*22q11.21*	c.34del	p.Leu12SerfsTer70	M	
29	*PHEX*	Small deletion	NM_000444.4	*Xp22.11*	c.917del	p.Ser306Metfs*3	M	OP
30	*PKD1*	Missense	NM_001009944.3	*16p13.3*	c.2534T>C	p.Leu845Ser	P	LB
31	*PKD1*	Missense	NM_001009944.3	*16p13.3*	c.8311G>A	p.Glu2771Lys	P	LB
32	*PKD1*	Missense	NM_001009944.3	*16p13.3*	c.1938G>A	p.Trp646Ter	P	LB
33	*PKD1*	Small deletion	NM_001009944.3	*16p13.3*	c.12494_12501del	p.Ser4165TrpfsX42	P	LB
34	*PKD1*	Missense	NM_001009944.3	*16p13.3*	c.6424C>T	p.Gln2142Ter	M	LB
35	*PKD1*	Small deletion	NM_001009944.3	*16p13.3*	c.9388_9393 del	p.Arg3130_Gly3131 del	M	LB
36	*PKD1*	Missense	NM_001009944.3	*16p13.3*	c.12031C>T	p.Gln4011*	M	LB
37	*PKD1*	Missense	NM_001009944.3	*16p13.3*	c.9547C>T	p.Arg3183*	P	OP
38	*PKD1*	Repeat variants	NM_001009944.3	*16p13.3*	c.786dup	p.Thr263HisfsTer108	M	Implantation failure
39	*PKD1*	Small deletion	NM_001009944.3	*16p13.3*	c.11241_11255del	p.Arg3750_Leu3754 del	P	Implantation failure
40	*PKD1*	Repeat variants	NM_001009944.3	*16p13.3*	c.7415dup	p.Ser2475LeufsTer26	M	Frozen
41	*PKD1*	Missense	NM_001009944.3	*16p13.3*	c.6132C>G	p.Asn2044Lys	M	Frozen
42	*PKD1*	Missense	NM_001009944.3	*16p13.3*	c.10821 + 1G>A	p.Leu3608Met	P	Frozen
43	*PKD2*	Missense	NM_000297.4	*4q22.1*	c.1081C>T	p.Arg361*/p.Arg361 Ter	M	Implantation failure
44	*PKHD1*	Missense	NM_138694.4	*6p12.3-p12.2*	c.11314C>T	p.Arg3772Ter	M	LB
	*PKHD1*	Small indels	NM_138694.4	*6p12.3-p12.2*	c.9235_9236 delinsAA	p.Ala3079Lys	P	
45	*PKHD1*	Missense	NM_138694.4	*6p12.3-p12.2*	c.11074C>T	p.Arg3692*	M	LB
	*PKHD1*	Missense	NM_138694.4	*6p12.3-p12.2*	c.5993T>C	p.Ile1998Thr	P	
46	*PKHD1*	Missense	NM_138694.4	*6p12.3-p12.2*	c.2914A>T	p.Lys972Ter	M	LB
	*PKHD1*	Missense	NM_138694.4	*6p12.3-p12.3*	c.9662C>T	p.Pro3221Leu	P	
47	*PKHD1*	Missense	NM_138694.4	*6p12.3-p12.2*	c.1139T>C	p.Phe380Ser	P	BPR
	*PKHD1*	Missense	NM_138694.4	*6p12.3-p12.2*	c.1639T>C	p.Cys547Arg	M	
48	*PKHD1*	Small insertion	NM_138694.4	*6p12.3-p12.2*	c.1023_1024 insACTG	P.Glu342ThrfsTer5	P	OP
	*PKHD1*	Missense	NM_138694.4	*6p12.3-p12.2*	c.2947T>C	p.Cys983Arg	M	
49	*PKHD1*	Missense	NM_138694.4	*6p12.3-p12.2*	c.8518C>T	p.Arg2840Cys	P	Frozen
	*PKHD1*	Gross deletion	NM_138694.4	*6p12.3-p12.2*	Exon19del	−	M	
50	*PKHD1*	Missense	NM_138694.4	*6p12.3-p12.2*	c.11074C>T	p.Arg3692*	P	Frozen
	*PKHD1*	Missense	NM_138694.4	*6p12.3-p12.2*	c.5935G>A	p.Gly1979Arg	M	
51	*PKHD1*	Missense	NM_138694.4	*6p12.3-p12.2*	c.103G>T	p.G35* /p.Gly35Ter	M	Frozen
	*PKHD1*	Missense	NM_138694.4	*6p12.3-p12.2*	c.5935G>A	p.Gly1979Arg	P	
52	*PLCE1*	Missense	NM_016341.4	*10q23.33*	c.5426T>C	p.Ile1809Thr	P & M	LB
53	*PLP1*	Repeat variants	NM_199478.3	*Xq22.2*	g.103010788-103232003dup	−	M	Frozen
54	*RPGRIP1L*	Missense	NM_015272.5	*16q12.2*	c.427C>T	p.Gln143Ter	M	LB
	*RPGRIP1L*	Missense	NM_015272.5	*16q12.2*	c.1351-11A>G	−	P	
55	*RPGRIP1L*	Missense	NM_015272.5	*16q12.2*	c.2122G>A	p.Gly708Ser	M	Implantation failure
	*RPGRIP1L*	Small deletion	NM_015272.5	*16q12.2*	c.1419-1421del	−	P	
56	*SRD5A2*	Missense	NM_000348.4	*2p23.1*	c.680G>A	p.Arg227Gln	P & M	LB
57	*TMEM67*	Missense	NM_153704.6	*8q22.1*	c.1645C>T	p.R549C	M	LB
	*TMEM67*	Missense	NM_153704.6	*8q22.2*	c.2434G>T	p.Glu812Ter	P	
58	*TMEM67*	Small deletion	NM_153704.6	*8q22.1*	c.296del	p.Lys99fs	M	Frozen
	*TMEM67*	Missense	NM_153704.6	*8q22.1*	c.1243G>A	p.Val415Met	P	
59	*TMEM67*	Missense	NM_153704.6	*8q22.1*	c.166G>T	p.Asp56Tyr	M	Frozen
	*TMEM67*	Missense	NM_153704.6	*8q22.1*	c.1388G>A	p.Arg463Gln	P	
60	*TSC1*	Repeat variants	NM_001362177.2	*9q34*	c.989dup	p.Gly331ArgfsTer7	M	Frozen
61	*VPS33B*	Missense	NM_018668.5	*15q26.1*	c.1594C>T	p.Arg532Ter	P & M	BPR
62	*Xq28*	Repeat variants	−	*Xq28*	g.152932475-153683298dup	−	M	Frozen
63	*Xq28*	Repeat variants	−	*Xq28*	g.152925133-153530814dup	−	M	Frozen
64	*Xq28*	Gross deletion	−	*Xq28*	g.154120000-154580000del	−	M	Frozen

GRCh37 (hg19); BP, biochemical pregnancy; LB, live birth; M, maternal; OP, ongoing pregnancy; P, paternal. paternal; According to HGVS Nomenclature, * denotes termination code (nonsense mutation).

### Disease Carrier Frequencies in Expanded Carrier Screening

Our reproductive department would propose that all couples who referred to PGT undertook ECS screening to minimize the risk of having children with additional recessive genetic illnesses. During the study period, we performed a total of 7,311 expanded carrier tests in individuals request PGT. Considering the P/LP variants for known monogenetic diseases, there were 12.19% (891/7,311) carriers of known genetic kidney diseases and syndromic disorders with renal phenotypes. The most frequently P/LP variants were reported in *SLC26A4* gene with a carrier rate of 2.38% (174/7,311), followed by *USH2A* accounted for 2.37% (173/7,311), *PKHD1* (1.29%, 94/7,311), *MMACHC* (1.00%, 73/7,311), *ETFDH* (0.85%, 62/7,311), and *CEP290* (0.53%, 39/7,311) (detailed in Supplementary Table 1).

### Clinical Cycles of Assisted Reproductive

The critical procedure, oocyte-retrieval was performed in all of 64 females who came for counseling after a planned process including genetic diagnosis, examination, assisted reproductive therapy if necessary and personalized decision making. As a result, each participant retrieved 13.4 ± 8.2 oocytes after 10.0 ± 2.0 days COH with 31.2 ± 11.7 mIU/mL gonadotrophin (Gn), undergoing 1-4 oocyte-retrieval cycle on average. With an average of 4, 220.2 pg/ml, the estradiol (E2)-peak varied from 211 to 12, 235 pg/ml. The metaphase II stage (MII) rate of oocytes was 82.0 ± 17.0% for each participant in terms of oocyte quality and the blastocyst formation rate was 56.8 ± 29.3% (Supplementary Table 5). In our cohort, the mean maternal age at oocyte-retrieval were 31.3 ± 4.1 years (range 24–42 years), including 15.6% (10/64) over 35 years old (Table 1).

### Analysis of Preimplantation Genetic Testing for Monogenic Disease Diagnostic

In the 64 cases with P/LP variants of known disease-causing genes for kidney disease, 344 embryos biopsy samples were analyzed, among which five failed to sequencing due to insufficient embryo DNA. There were 65.4% of blastocyst being a euploid, whereas 29.9% of blastocyst being an aneuploid. In total, 31.6% (71/225) of the euploid embryos were unaffected, 33.3% (75/225) of the embryos were affected, 35.1% (79/225) of the embryos were carrier. There were 3.2% (11/344) low-level mosaicism (≤ 50%) were identified which could be the secondary candidates of FET if they also did not carry the disease-causing gene (Supplementary Table 2). The frequency of euploid embryos was calculated as 66.3% (193/291) and 60.4% (32/53) in maternal age group of <35 years and ≥35 years, respectively. As for the aneuploid embryos, the frequency accordingly was 28.2% (103/291) and 39.6% (21/53) in each age group, respectively (*P* > 0.05, Supplementary Table 2). Between 2011 and 2021, each couple who underwent the PGT-M experienced 1.30 ± 0.53 cycles of PGT-M test with at least one cell biopsy. A total of 339 (98.5%) embryos were successfully analyzed, 150 (43.6%) of them were transferable and to date 63 (18.6%) was transferred. The average transferable embryos were 0.9 ± 0.4 per oocyte-retrieval cycles and 2.2 ± 1.6 per PGT-M test cycles, respectively. In 64 cases with oocyte-retrieval, the cumulative rate of obtained transferable embryos for each couple was 85.9% (Supplementary Table 6).

### Pregnancy Outcome

There were 61 FET cycles underwent in the 43 couples with high risk of genetic kidney disease in our cohort. Single blastocyst was most frequently transferred (59/61) and a total of two double blastocyst transfers were performed. There were no monozygotic or dizygotic twins in any of the successful pregnancies. The chemical pregnancy rate, as defined by a positive beta hCG level, was 67.21% (41/61). The implantation rate, as defined by the presence of a gestational sac was 59.02% (36/61) and presence of fetal heartbeat was 57.38% (35/62). It should be noted that one of the couples with double blastocyst transferred had experienced SAB. Therein one blastocyst with no HB and the other blastocyst had a live birth. The biochemical pregnancy and spontaneous abortion rates were 9.84% (6/61) and 1.64% (1/61), respectively (Table 3).

**TABLE 3:** Pregnancy outcomes in 43 couples with high risk of genetic kidney disease.

Characteristics	Total = 61 FET cycles	Female age at IVF-FET < 35 years (*n* = 55)	Female age at IVF-FET ≥ 35 years (*n* = 6)	*P*-value
PHCG	67.21% (41/61)	67.27% (37/55)	66.67% (4/6)	1.000
IR	59.02% (36/61)	58.18% (32/55)	66.67% (4/6)	1.000
HB	57.38% (35/61)	56.36% (31/55)	66.67% (4/6)	1.000
BPR	9.84% (6/61)	10.91% (6/55)	0.00% (0/6)	0.394
SAB	1.64% (1/61)	0.00% (0/55)	16.67% (1/6)	0.098
OP/LBR	57.38% (35/61)	56.36% (31/55)	66.67% (4/6)	1.000

*BPR, biochemical pregnancy; FET, frozen-embryo transfer; HB, fetal heartbeat; IR, implantation rate; IVF, in vitro fertilization; OP/LBR, ongoing pregnancy/live birth rate; PHCG, positive-HCG; rate; SAB, spontaneous abortion.*

In the 61 cycles of FET for genetic kidney disease, ongoing pregnancy (OP)/live birth rate (LBR) reached 57.38% (35/61). And the cumulative OP/LBR in our cohort was 54.69% (35/64) by the end of 2021 (Figure 3B). The follow-up rate of amniotic fluid was 100%, which was consist with PGT-M (data not shown). The mean gestation week was 38.9 weeks. And the mean birth weight was 3530.6 ± 423.1 g. There is no significant difference in the gender (boys vs. girls: 5:4) of the babies. During the follow-up in our medical centers with pediatrics and consultants of nephrology, no neonate malformations or any condition associated with renal disease were reported.

## Discussion

In the current study, we presented the clinical outcome of kidney-related PGT-M performed in a single medical center over the past 10 years. A cumulative ongoing pregnancy/live birth rate 54.69% by the end of 2021 were achieved in our cohort with risk of genetic kidney disease. It highlighted the necessity of molecular genetic diagnosis for kidney disease and the importance of reproductive counseling for the couples with potential risk of kidney disease.

Preimplantation genetic testing entails genetic testing of embryos obtained through IVF to screening out the genetic disorders. Only embryos that are free of the disorders are then suitable for implantation. According to a report from the Netherlands based on a 25-year history of using PGT to prevent the offspring with kidney disorders, two-thirds achieved at least one live birth rate, which was comparable to IVF outcomes in general (3). The majority of couples in this retrospective cohort had AD or X-linked disease, with the mother more being the affected parent than the father. Among the 537 embryos for biopsy from the 52 couples, 35% were free of the genetic kidney disease and suitable for transfer (3). Here, our PGT cohort from 2011 to 2021 provided the number of kidney-related PGT-M involved in analyzing of the 64 couples and 344 embryos with 20.6% unaffected euploid embryos and 150 (43.6%) were free of the genetic kidney disease and suitable for transfer. The cumulative ongoing pregnancy/live birth of 54.69% was acceptable and better compared to the previous report of Dutch cohort (3). The average maternal age of 30.6 years at FET, which was younger than the Dutch cohort (32 years old) might have contributed to the higher ongoing pregnancy/live birth. It has been demonstrated that women <35 years old had significantly higher euploid blastocyst rates when compared women >35 years old (24). More blastocysts to biopsy and vitrify means more euploid blastocysts to choose from in the corresponding FET cycle for the young participants. In addition, the MII rate (82.0%) and blastocyst formation rate (56.8%) in our center were at the leading level, which made enough blastocysts available for analysis and implantation. Unfortunately, there was no detail information on the blastocyst euploidy and implantation rates from the Dutch cohort (3). Furthermore, the trend of higher incidence of aneuploidy in the group over 35 years old compared with that under 35 years old was in line with expectations, but not statistically significant. It may be due to that the aged female in this cohort accounted for a small proportion and the average age was relatively young. It was worth exploring with a larger sample size as the growing consciousness of the advantages of PGT-M in the future.

Underlying the genetic findings of kidney-related PGT is crucial for the parents at risk for passing a genetic condition to their children, especially when faced with the difficult decision of pregnancy termination if the fetus has been diagnosed with congenital abnormalities generally in the second trimester. During the study period, P/LP variants of known disease-causing genes for kidney disease were identified in 13.8% of the total cases of PGT-M referred to our medical center. In our cohort, 54.7% of the couples had AR genes, 29.7% had AD genes and 15.6% had XL genes. Among the 38 genetic kidney diseases, PKD was the most common one, followed by NPHP, metabolic disease and CAKUT. A report from a commercial laboratory detailed the experience with kidney-related PGT-M in 389 cases referred from 98 different IVF clinics across America between 2010 and 2019 (24). In the American cohort, 52% of couples referred for AR genes, 32% were screened for AD genes and 14% for XL inheritance (24). Comparing with the gene spectrum presented from Dutch cohort (3), American cohort (6), and our Chinese cohort, PKD and Alport syndrome were the most common diseases referred for PGT. Furthermore, we presented more cases with genetic kidney disease which have not been published in the literature of the experience on PGT such as congenital nephrotic syndrome caused by *PLCE1* or *NHPS1*, and NPHP caused by *NPHP1, TMEM67*, or *MKS1 et al*.

A growing variety of preconception carrier tests for genetic disease are now available for couples planning to conceive. Initially, carrier testing for AR disease was conducted for genes that were frequent in high risk population for certain inherited disease groups (25). The chance of being a carrier for a genetic disease is dependent on ethnicity and family history, with certain populations having a higher baseline incidence of certain condition (26). However, *de novo* variants can also occur, and genetic condition is not isolated within a certain community. As is well-known the majority of genetic kidney disease is AR disease, ECS for assisted conception couples combined with PGT is necessary for reproductive counseling. Our results showed the effectiveness of the ECS prior to PGT cycle with a carrier rate of 12.2% in known genes for monogenetic kidney diseases and syndromic disorders with renal phenotypes. Offering ECS to couples with family history of kidney disease would seem preferable, whereas couples who are unaware of the risk of genetic kidney disease carriers will confront a difficult decision-making process. Genetic kidney diseases are the fourth most common cause of renal failure in the world. Many kidney diseases such as ARPKD, Alport syndrome, or NPHP may be diagnosed until the development of renal failure during adolescence or adult stage. In general, the implementation of PGT-M relies heavily on prior specialist molecular diagnosis. For CAKUT, as we knew, less to 50% of the patients can be identified carrying genetic background (27). For the parents with this child, it is much difficult for PGT-M than other genetic kidney disease such as polycystic kidney disease. Prioritizing the embryo transfer order based on the information of PGT and ECS data (ranking), is expected to minimized the risk of an adverse pregnancy outcome (biochemical pregnancy, clinical miscarriage, and artificial abortion).

There were some limitations to current study. First, our cohort was from a single center, which was not population-based. However, the center is one of the largest IVF and PGT center in China, involving couples from various regions, thus diminishing the regional bias. Secondly, further work on the long-term follow-up is to carried out to analyze the periconceptional effect on clinical outcomes of PGT-M. However, there were no records of spontaneous pregnancy or PGT-M misdiagnoses among our cohort. In addition, cases of sperm and egg donation in various forms to reduce the risk of having children with genetic kidney disease were not included in this retrospective study (28). Fertility preservation for women with genetic kidney disease wishing to conceive is also not involved in our case (29, 30). The applied range of PGT-M at the present stage still has limitations in some specific situation in our center. For example, PGT-M refers to testing for nuclear DNA pathogenic variant(s) exclusion testing and disorders caused by pathogenic variants in the mitochondrial DNA (mtDNA). Nuclear transfer has been applied to minimize transmission risk of mtDNA diseases. However, it is not allowed in China for ethical reasons and some patients who came to our IVF center for consultation would choose to reduce the risk through egg donation. Furthermore, for special cases, including *de novo* (31) and germline mosaicism (32) pathogenic variants in husband or wife, PGT-M currently adopts the strategy of constructing haploid for linkage analysis by SNPs through next-generation sequencing (NGS) or Karyomapping array combined with direct sequencing methods such as Sanger sequencing. Our center has carried out relevant cases and achieved successful healthy live births. For *de novo* variants, direct detection methods such as Sanger sequencing are used to find variant carriers in sperm, polar bodies or blastocysts as probands. For germline mosaicism (32) pathogenic variants, embryos that the linkage analysis showed carrying allele different from the diseased proband and the Sanger sequencing did not detect the pathogenic variant were recommended as priority embryos for transfer; embryos that the linkage analysis showed carrying allele same as the diseased proband but the sanger sequencing did not detect the pathogenic variant were recommended as secondary transfer embryos.

In conclusion, we provided the overview of PGT referrals for genetic kidney disease over the ten-year study from one medical center in China. Due to the advance in genetic kidney disease, there has been an increase of kidney-related PGT referral since 2018. In our cohort, the high rate of unaffected live born children following PGT in monogenic kidney disease can support the counseling for families at risk of kidney disease. As the need for decision-making assistance for prospective parents and appropriate referrals to reproductive specialists grows, awareness of PGT as a reproductive option for couples among the nephrology community is crucial.

## Data Availability Statement

The datasets presented in this article are not readily available due to patient privacy and confidentiality. Requests to access the datasets should be directed to to XS, xiaoxi_sun@aliyun.com.

## Ethics Statement

The studies involving human participants were reviewed and approved by the Medicine Ethics Committee of Children’s hospital, Fudan University (NO. 2018_286). The patients/participants provided their written informed consent to participate in this study.

## Author Contributions

MX, HS, and JR completed the main data analysis and manuscript writing. YX was mainly responsible for the analysis of ECS data. CL and JW performed genetic counseling of patients. XS was responsible for the IVF process of our center. JR and HX were responsible for nephropathy diagnosis in this cohort. MX and ShZ analyzed bioinformatics data of PGT. SaZ and JZ performed preimplantation genetic testing (PGT) experiments. All authors contributed to the article and approved the submitted version.

## Conflict of Interest

XS was the guarantor of this work, and as such, had full access to all the data in the study and took responsibility for the integrity of the data and accuracy of the data analysis. The remaining authors declare that the research was conducted in the absence of any commercial or financial relationships that could be construed as a potential conflict of interest.

## Publisher’s Note

All claims expressed in this article are solely those of the authors and do not necessarily represent those of their affiliated organizations, or those of the publisher, the editors and the reviewers. Any product that may be evaluated in this article, or claim that may be made by its manufacturer, is not guaranteed or endorsed by the publisher.

## Footnotes

1
www.illumina.com

2
www.mlpa.com

## Abbreviations

AD: autosomal dominant
AR: autosomal recessive
ART: assisted reproductive technology
ACMG: the American College of Medical Genetics and Genomics
BPR: biochemical pregnancy rate
CAKUT: congenital anomalies of the kidney and the urinary tract
CKD: chronic kidney disease
CNV: copy number variations
COH: controlled ovarian hyperstimulation
CSA: clinical sequence analyser
E2: estradiol
ECS: expanded carrier screening
ESRD: end-stage renal disease
FHB: fetal heartbeat
FETs: frozenembryo transfers
GnRH: gonadotrophic releasing hormone
HGVS: human genome variation society
hCG: human chorionic gonadotropin
ICSI: intracytoplasmic sperm injection
IR: implantation rate
IVF: *in vitro* fertilization
MII: metaphase II stage
NGS: next-generation sequencing
NS: nephrotic syndrome
NPHP: nephronophthisis
OP/LBR: ongoing pregnancy/live birth rate
PGT: preimplantation genetic testing
PGT-A: PGT for aneuploidies
PGT-SR: PGT for structural rearrangements
PGT-M: preimplantation genetic testing for monogenic disease
PHCG: positive-human chorionic gonadotropin
PKD: polycystic kidney disease
PND: invasive prenatal diagnosis
TE: trophectoderm
SAB: spontaneous abortion
SNP: single nucleotide polymorphism
WES: whole exome sequencing
WGS: whole genome sequencing.
