# Supplementary material for: Combined Preimplantation Genetic Testing for Genetic Kidney Disease: Genetic Risk Identification, Assisted Reproductive Cycle, and Pregnancy Outcome Analysis
Source: Front Med (Lausanne). 2022 Jun 17;9:936578. doi: 10.3389/fmed.2022.936578 (PMC9247246; doi:10.3389/fmed.2022.936578)
Supplement: Supplementary file 1 [file Data_Sheet_1.pdf]

Supplementary Material

Supplementary Table 1. Carrier frequencies of monogenic kidney diseases and genes in 7,311 Han ethnicity individuals.

**Genes**	**Diseases**	**n of positive cases**	**positive rate (total n = 7,311)**
SLC26A4	Pendred Syndrome	174	2.38%
USH2A	Usher Syndrome, Type 2a	173	2.37%
PKHD1	Autosomal Recessive Polycystic Kidney Disease	94	1.29%
MMACHC	Methylmalonic Aciduria and Homocystinuria, Cblc Type	73	1.00%
ETFDH	Multiple Acyl-Coa Dehydrogenase Deficiency	62	0.85%
CEP290	Joubert Syndrome	39	0.53%
COL4A3	Alport syndrome	36	0.49%
AGXT	Primary Hyperoxaluria	34	0.47%
PEX1	Zellweger Syndrome	31	0.42%
AHI1	Joubert Syndrome	28	0.38%
NPHS1	Nephrotic Syndrome, Type 1	25	0.34%
DHCR7	Smith-Lemli-Opitz syndrome	20	0.27%
BBS2	Bardet-Biedl Syndrome 2	15	0.21%
CPT2	Carnitine Palmitoyltransferase II Deficiency	12	0.16%
CYP17A1	Congenital Adrenal Hyperplasia Due to 17-Alpha-Hydroxylase Deficiency	11	0.15%
GRHPR	Primary Hyperoxaluria Type 2	10	0.14%
NPHS2	Nephrotic Syndrome, Type 2	9	0.12%
CTNS	Nephropathic Cystinosis	8	0.11%
FANCC	Fanconi Anemia Complementation Group C	8	0.11%
BBS1	Bardet-Biedl Syndrome 1	7	0.10%
COL4A4	Alport syndrome	6	0.08%

BBS10	Bardet-Biedl Syndrome 10	4	0.05%
ETFA	Multiple Acyl-Coa Dehydrogenase Deficiency	3	0.04%
GLA	Fabry Disease	3	0.04%
PEX6	Zellweger Syndrome	3	0.04%
ETFB	Multiple Acyl-Coa Dehydrogenase Deficiency	2	0.03%
CYP11B1	Congenital Adrenal Hyperplasia Due to 11-Beta-Hydroxylase Deficiency	1	0.01%
**Total**		891	12.19%

Supplementary Table 2. Frequency of euploid, aneuploid, or mosaicism in PGT-M.

Characteristics	Total	Female age at IVF-FET < 35 years (n =291)	Female age at IVF-FET≥35 years (n =53)	*P* value
**Euploid**	65.4% (225/344)	66.3% (193/291)	60.4% (32/53)	0.403
Unaffected	31.6% (71/225)	31.6% (61/291)	31.3% (10/53)	0.968
Affected	33.3% (75/225)	31.1% (60/291)	46.9% (15/53)	0.079
Carrier	35.1% (79/225)	37.3% (72/291)	21.9% (7/53)	0.090
**Aneuploid**	29.9% (103/344)	28.2% (82/291)	39.6% (21/53)	0.094
**Low-level mosaicism**	3.2% (11/344)	3.8% (11/291)	0.0% (0/53)	0.150
**Low DNA concentration**	1.5% (5/344)	1.7% (5/291)	0.0% (0/53)	1.000

PGT-M: preimplantation genetic testing for monogenic disease; Mosaicism refers to includes whole-chromosome and CNV-level mosaicism. The low-level mosaicism refers to the proportion of abnormal mosaicism was less than 50% and the simple low-level mosaicism could be the secondary candidates of frozen-embryo transfer (FET); IVF: *in vitro* fertilization

Supplementary Table 3. Genetic Spectrum of Consultants.

Classification	Gene	n	%
NS	*NPHS1*	1	1.6
	PLCE1	1	1.6
CAKUT	*Xq28*	3	4.7
	PAX2	1	1.6
	Xq22.2	1	1.6
PKD	*PKD1*	13	20.3
	PKHD1	8	12.5
	PKD2	1	1.6
Alport Syndrome	*COL4A3*	1	1.6
	COL4A4	1	1.6
	COL4A5	1	1.6
NPHP	*TMEM67*	3	4.7
	NPHP1	1	1.6
	NPHP4	1	1.6
	BBS7	1	1.6
	CPLANE1	1	1.6
	CC2D2A	1	1.6
	MKS1	1	1.6
	RPGRIP1L	2	3.1
	OFD1	1	1.6
Metabolic Syndrome	*MMACHC*	1	1.6
	GLA	1	1.6
	ALPL	1	1.6
	HPRT1	1	1.6
	MMUT	1	1.6
	PCCA	1	1.6
	PCCB	1	1.6
	PHEX	1	1.6
Others	*PEX1*	2	3.1
	DYNC2H1	2	3.1
	H19	1	1.6
	FAT4	1	1.6

	LMX1B	**1**	**1.6**
	MAMLD1	**1**	**1.6**
	PEX26	**1**	**1.6**
	SRD5A2	**1**	**1.6**
	TSC1	**1**	**1.6**
	VPS33B	**1**	**1.6**

CAKUT: congenital anomalies of the kidney and the urinary tract; NS: nephrotic syndrome; NPHP: nephronophthisis; PKD: polycystic kidney disease.

Supplementary Table 4. Genetic characteristics

Features	
**Genetic characteristics**	**n of couples (%)**
Autosomal dominant disease	19 (29.7)
Autosomal recessive disease	35 (54.7)
X-linked disease	10 (15.6)
**In case of autosomal dominant or X-linked disease**	**n of total cohort (%)**
Genetically affected parent is the father	9 (14.1)
Genetically affected parent is the mother	21 (32.8)

Supplementary Table 5. Oocyte-retrieval data of patients undergoing PGT-M.

Features	Total couples, n=64		
	**mean±SD**	**min**	**max**
Number of cycles to oocyte retrieval (per couples)	1.4±0.7	1	4
Duration of Gn (Days)	10.0±2.0	6	20
Dosage of Gn (mIU/mL)	31.2±11.7	12	60
E2-peak (pg/ml)	4, 220.2±2, 641.5	211	12, 235
Endometrial thickness (mm)	10.3±2.8	4	19
Oocyte acquired	13.4±8.2	0	39
MII rate (%)	82.0±16.7	0.3	1
Oocytes fertilized	10.9±7.1	0	39
Number of blastocysts cultured	6.7±4.9	0	22
Number of frozen embryos	3.9±3.3	0	13
The blastocyst formation rate (%)	56.8±29.3	0.0	100%

E2: estradiol; Gn: gonadotrophin; MII: metaphase Ⅱ stage

Supplementary Table 6. Cycles of PGT-M test and FET data of patients undergoing PGT-M.

Features	Total embryos, n=344
	**n**
Cycles to PGT-M test	78 (1.3±0.5, per couples)
Cycles with one biopsy	77(98.7%)
Cycles with two biopsies	1(1.3%)
Embryos diagnosed	339 (98.5%)
Transferable embryos	150 (43.6%, 2.3±1.6 per couples)
Transferable embryos per PGT-M test cycles	2.2±1.6
Transferable embryos per oocyte-retrieval cycles	0.9±0.4
Cumulative transferable rate	85.9%
Embryos transferred	63 (18.3%)
Cycles to embryo transfer	61

FET: frozen-thawed embryo transfer; PGT-M: preimplantation genetic testing for monogenic disease

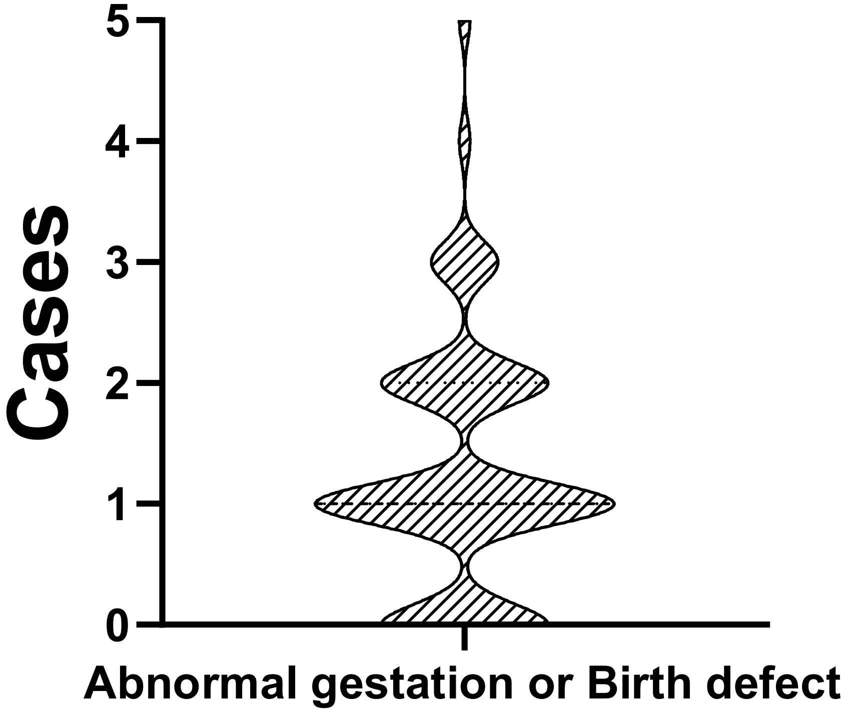

Supplementary Figure 1. Pregnancy History of the Consultants. The distribution of the pregnancy history aggregately. Most couples had experienced 1 or 2 times of abnormal gestations or birth defects, while some couples had experienced 4 to 5 times of abnormal gestations and birth defects before counseling.
